# Monocular and Binocular Contributions to Oculomotor Plasticity

**DOI:** 10.1038/srep31861

**Published:** 2016-08-18

**Authors:** Guido Maiello, William J. Harrison, Peter J. Bex

**Affiliations:** 1 UCL Institute of Ophthalmology, University College London, 11-43 Bath Street, London EC1V 9EL, UK; 2 Department of Psychology, Northeastern University, 360 Huntington Ave., Boston, Massachusetts 02115, USA; 3 Department of Psychology, University of Cambridge, Free School Lane, Cambridge, CB2 3RQ, UK; 4 Queensland Brain Institute, The University of Queensland, St Lucia QLD 4072, Australia

## Abstract

Most eye movements in the real-world redirect the foveae to objects at a new depth and thus require the co-ordination of monocular saccade amplitudes and binocular vergence eye movements. Additionally to maintain the accuracy of these oculomotor control processes across the lifespan, ongoing calibration is required to compensate for errors in foveal landing positions. Such oculomotor plasticity has generally been studied under conditions in which both eyes receive a common error signal, which cannot resolve the long-standing debate regarding whether both eyes are innervated by a common cortical signal or by a separate signal for each eye. Here we examine oculomotor plasticity when error signals are independently manipulated in each eye, which can occur naturally owing to aging changes in each eye’s orbit and extra-ocular muscles, or in oculomotor dysfunctions. We find that both rapid saccades and slow vergence eye movements are continuously recalibrated independently of one another and corrections can occur in opposite directions in each eye. Whereas existing models assume a single cortical representation of space employed for the control of both eyes, our findings provide evidence for independent *monoculomotor* and *binoculomotor* plasticities and dissociable spatial mapping for each eye.

Humans and many animals with overlapping binocular visual fields continuously shift their gaze point in three-dimensional (3D) space in order to bring targets of interest onto the high-resolution fovea or area centralis of the two eyes. These gaze shifts require highly precise and coordinated fast saccadic and slow vergence eye movements. To achieve binocular coordination in a 3D environment, Hering^1^ proposed that both eyes are innervated by conjugate command signals. Alternatively, Helmholtz^2^ argued that binocularly coordinated visual systems evolved from independently moving lateral eyes and that each eye is controlled independently^3^ (for a review see^4^). The finding that some saccadic eye movements in 3D space result in each eye moving by a different amount^5^,^6^ has generally been seen as a challenge to Hering’s law of equal innervation. However, it has been argued these data may yet be explained by a vergence command superimposed on a conjugate saccadic command^7^.

In order to maintain the accuracy and precision of eye movements in 3D environments over time, oculomotor control processes must also be capable of adapting to lifespan changes that affect the forces acting on each eye. For example during normal aging, extraocular muscles become displaced^8^, loss of structural support leads to orbital fat prolapse^9^, the area of the orbital aperture increases and the eye sockets become wider and longer^10^. The plasticity of the oculomotor control system has been widely investigated with short-term saccade adaptation paradigms^11^. In a double-step saccade adaptation study, an observer is asked to make a saccadic eye movement to a target. During the saccade, the target is displaced so that the observer’s saccade fails to bring their fovea to the target. Due to saccade masking^12^,^13^,^14^,^15^ and poor sensitivity to high speed retinal images^16^,^17^, the intra-saccadic displacement is not noticed by the observer. Consequently, when the eyes land, there is an apparent sensorimotor mismatch between the actual foveal signal and the foveal signal expected given the executed eye movement^18^. Over the course of a few dozen trials, the oculomotor system recalibrates, altering the saccade gain so as to reduce the mismatch on subsequent trials (for a recent review see^19^). Analogous saccadic adaptation processes could in principle account for longitudinal oculomotor plasticity during aging. Additionally, in order to maintain accurate binocular calibration when aging processes around each eye occur asymmetrically, saccade adaptation would have to operate independently for each eye. However, it is not currently known whether oculomotor adaptation can occur separately for each eye, since adaptation is usually studied with identical error signals in both eyes. One study that investigated monocular adaptation showed that adaptation induced only in one eye transfers to the other eye^20^, which would suggest that saccade adaptation may not be capable of operating independently for each eye.

In this study, we employed the classic short-term double step saccade adaptation paradigm^11^ to induce saccade recalibration in six normally-sighted young adults. We modified this paradigm to test how the oculomotor system adapts to different errors in the saccade end points of the two eyes. We compared oculomotor responses to conjugate saccade adaptation (i.e. same error in both eyes) with oculomotor responses to dichoptic saccade adaptation (i.e. different error in the two eyes). By studying asymmetric monocular oculomotor adaptation, we addressed two outstanding questions in visual neuroscience. Firstly, we examine whether eye movement control is conjugate for both eyes, as originally argued by Hering^1^, or independent for each eye, as originally suggested by Helmholtz^2^. If we find asymmetric adaptation, this would provide convincing evidence in favour of independent monocular innervation. Secondly, we examine whether independent saccade adaptation could provide the basis for oculomotor calibration for asymmetric changes on the forces of the eyes across the lifespan.

The experimental conditions are illustrated in Fig. 1. Observers executed a saccade to track a Gabor target (σ = 0.5°, ω = 4 cycles/degree) that stepped from the centre of the monitor to a position at 8° to the left (Fig. 1a) or right. During the saccade, the target stepped invisibly a second time by 10% (0.8°) of the initial displacement. As shown in Fig. 1b, the secondary target step was either outward in both eyes (outwards step), inward in both eyes (inwards step), or outward in the temporally moving eye and inward in the nasally moving eye (dichoptic step). For the outward and inward step conditions, we expect observers’ saccade gain to increase and decrease, respectively^19^. It is currently unknown how the oculomotor system will respond to the dichoptic steps.

## Results

For each target-step condition, Fig. 2 shows changes in saccade amplitude (y-axis) across adaptation trials (x-axis). In all plots, red and blue data show saccade amplitudes for the temporally and nasally moving eye, respectively, collapsed across subjects and eye movement direction. In good agreement with previous studies^19^, in the outward step condition (Fig. 2a), the saccades of both eyes increased in amplitude across adaptation trials; and in the inward step condition (Fig. 2b), the saccades of both eyes decreased in amplitude. Critically, however, in the dichoptic step condition (Fig. 2c), the saccades executed by the temporally moving eye (red curve) increased in size, while the saccades in the nasally moving eye (blue curve) decreased in size. Therefore, when presented with opposing error signals, the eyes adapt in different directions. It is worth noting however, that whereas in the early adaptation trials of the dichoptic step condition the saccade amplitudes indeed vary in different directions disconjugately, in the late adaptation the saccades executed by both temporally and nasally moving eyes appear to increase in size together, conjugately.

To quantify these observations, we fit the saccade amplitude data with second-degree polynomial (parabolic) equations, and extracted from these fits quantitative estimates of the adaptation dynamics (see *Materials and Methods* for a detailed description of the analytical procedures). The initial gain of adaptation, given by the declivity parameter of the fit, is shown in Fig. 2d. Note that the declivity parameter is independent of any baseline differences in saccade amplitudes across eyes and conditions. Adaptation gain increased for the outward step condition and decreased for the inward step condition (difference between outward and inward step conditions: p = 0.000051). In both these conditions, there was no significant difference in the adaptation gain between the temporally moving and nasally moving eyes (both p = 0.99). Conversely, in the dichoptic step condition, adaptation gain increased in the temporally moving eye and decreased in the nasally moving eye (difference between the temporally and nasally moving eye: p = 0.036). These data show that the saccades in each eye were independently recalibrated in opposite directions according to the different error signal simulated in each eye.

Immediately after each run of adaptation trials, observers completed a recovery procedure in which they performed the same saccade task but without the intra-saccadic target step. This was completed to observe the dynamics and ensure recovery from the transient eye deviations induced during adaptation. Note that relative to the adapted state of the observer, the change during recovery is inward in the temporal eye and outward in the nasal eye and thus examines the opposite direction of adaptation (convergent adaptation). Recovery patterns were the reverse of adaptation patterns for all three conditions (see Supplementary Data), confirming that separable and reversible adaptation had occurred in each eye.

In principle, the dichoptic adaptation described above could be attributable to independent monocular adaptation of the saccadic system for each eye, or binocular adaptation of vergence eye movements, as has been shown in the monkey^21^. To differentiate between these competing accounts, we examined inter-ocular divergence around the time of saccades (Fig. 3). Figure 3a shows inter-ocular divergence (y-axis) as a function of time from saccade onset (x-axis) for the dichoptic step condition. During the first ten adaptation trials (green shaded region) all observers executed vergence eye movements following the saccades to foveate the dichoptic post-saccadic target. This vergence response (green curve) was initiated (green star) ~100 ms after the saccade had ended. Were the changes in amplitude in the dichoptic step condition (Fig. 2c) driven purely by vergence adaptation, this vergence function should shift leftward across trials. However, in contrast to the continuous vergence function for the first ten adaptation trials, the last ten adaptation trials (red shaded region) included two components. For all six subjects, first there was a rapid divergence (ranging from 0.1° to 0.5° across subjects) which occurred during the saccade (red shaded region overlapping the grey shaded region in Fig. 3a), consistent with differential adaptation of saccade amplitudes in each eye. Only following this saccadic component is there a second vergence component, beginning after the end of the saccade (red star). These vergence eye movements also underwent adaptation, as revealed by their decreasing latency throughout the experiment (Fig. 3b). Similarly, the amplitude of the post-saccade vergence response diminished throughout the adaptation session (Fig. 3c). Thus, vergence eye movements were modified during the dichoptic adaptation procedure, independently of saccadic amplitudes.

## Discussion

We investigated monocular and binocular contributions to oculomotor plasticity. We replicated classic conjugate saccade adaptation results^11^: an invisible intra-saccadic binocular target shift leads to an automatic adaptation of the saccade amplitude in both eyes. Using a dichoptic saccade paradigm we further showed that when the intra-saccadic displacement is in opposite directions in each eye (dichoptic step condition), adaptation occurs in opposite directions in each eye. Conversely, after removing the dichoptic step in the recovery trials, we found the direction of adaptation reversed in both eyes, revealing convergent adaptation. Therefore, our data strongly suggest that our eyes receive at least partially independent recalibration commands. In the dichoptic step condition however, following an initial stage of apparently independent disconjugate adaptation we also qualitatively observed some degree of conjugate adaptation, i.e. saccade amplitudes in both eyes appeared to increase in size together.

Our results therefore inform the unresolved^4^ debate between Hering^1^ and Helmholtz^2^ regarding the nature of binocular oculomotor control. We show that binocular adaptation involves multiple potentially competing processes: independent disconjugate adaptation of monocular saccades, conjugate adaptation of binocular saccades, and binocular adaptation of vergence eye movements. Collectively, these adaptation processes likely serve to maintain binocular coordination while ensuring that the foveae of both eyes are directed to the intended locations of saccade targets. This plasticity could maintain saccade accuracy in the face of changes in the forces acting upon the eye during normal development and aging (for a review see ref. 9). Our data also show that it may be possible to modify binocular coordination via oculomotor plasticity. This result informs our understanding of the development of normal binocular eye movement control in children^22^,^23^ and has potentially significant implications for rehabilitation of oculomotor dysfunctions, including strabismus and vergence insufficiency.

Recent reviews of the literature have contrasted Helmholtz against Herring on theories of binocular eye movement coordination. These reviews have found evidence in favour and against both Herring’s and Helmholtz’s theories^4^ and suggest the controversy might be based on a false dichotomy^24^. Premotor neurons in the paramedian pontine reticular have been found to encode monocular eye movements^3^, yet it is possible that signals from these monocular neurons are combined downstream to produce binocular innervation of the eyes^25^,^26^. Both monocular and binocular control networks likely coexist and cooperate to produce eye movements in depth. In this framework, recalibration mechanisms might act at multiple stages of the oculomotor control process and produce a complex pattern of adjustments. Independent adaptation of monocular saccades, conjugate adaptation of binocular saccades, and binocular adaptation of vergence could reflect different neural recalibration loci. This hypothesis is further supported by previous and apparently conflicting literature. Albano and Marrero have shown that adaptation induced only in one eye transfers to the other, occluded, eye^20^, suggesting that recalibration was acting at the level of binocular innervation. In the same study however Albano and Marrero also observed that when both eyes were unoccluded and an error signal was simulated in one eye only, adaptation only partially transferred to the other eye, a result we have recently replicated^27^ and which suggest a combination of both monocular and binocular complimentary recalibration mechanisms. Independent monocular recalibration processes are also consistent with data from macaca fascicularis monkeys in which monocular surgical weakening of extraocular muscles produces rapid monocular recalibration without the involvement of vergence adaptation^28^. Conversely, Schultz and Busettini have recently observed binocular recalibration of the saccadic system via a mechanism which acts solely on the vergence system^21^.

Our results might also identify further differences in oculomotor control between human and other primates. Our data on classic conjugate saccade adaptation confirm that adaptation in humans can occur in tens of trials, whereas in monkeys adaptation takes several hundred trials^29^. In rhesus monkeys, changes in saccade amplitude following dichoptic post-saccadic errors were argued to derive exclusively from vergence adaptation superimposed on a common saccadic command^21^. In humans, we find evidence of both rapid, independent monocular saccade adaptation together with a slower binocular vergence adaptation, consistent with evidence suggesting that humans have distributed neural adaptation loci yet monkeys have a common site of adaptation^29^. It is however possible that monocular adaptation occurs in monkeys as well as humans, and that the existing analyses, which focus on highlighting binocular vergence adaptation (see Fig. 9 of ref. 21), mask the existence of independent monocular saccade adaptation in monkeys.

Our study highlights how understanding oculomotor recalibration in depth, which has scarcely been investigated in the literature, will help to map out and understand the neural control of binocular eye movements. Mapping out the resolution of oculomotor recalibration in 3D space^30^, as well as separating out the individual saccadic and vergence^31^ based components of oculomotor recalibration, will allow for a more rigorous testing of more detailed models of eye movement control. To the best of our knowledge, the literature on eye movement control does not discuss the possibility of dissociable spatial maps for each eye, and it is generally assumed that a single distributed cortical representation of space is employed for the control of both eyes^32^,^33^,^34^,^35^,^36^. Our findings raise the possibility that the oculomotor system maintains dissociable spatial maps for each eye or independently accesses a common map for both eyes.

## Materials and Methods

### Participants

Two authors (GM and WJH) and five naïve observers (all male, ages ranging from 20 to 32 years) were recruited for the study. All subjects had normal or corrected to normal vision. Six out of the seven subjects had had normal stereo vision, as confirmed by a stereoscopic Vernier acuity task (see Supplementary Experimental Procedures) performed prior to the main experiment. One subject had mild amblyopia, which was detected by the Vernier acuity task, and was excluded from further testing. All procedures were approved by the Northeastern University Institutional Review Board and adhered to the tenets of the declaration of Helsinki. All methods were carried out in accordance with the approved guidelines. All subjects provided written informed consent.

### Stimuli and Apparatus

All experiments were programmed with the Psychophysics Toolbox Version 3 37,38 and Eyelink Toolbox^39^ in Matlab (MathWorks). Eye-tracking was performed using the EyeLink 1000 (SR Research) desktop mount eye-tracker. The eye tracker was calibrated binocularly using the native thirteen-point calibration routine at the start of each experiment. Eye-tracking data was recorded at 500 Hz per eye and stimuli were presented on an ASUS VG278HE LCD monitor with a resolution of 1920 × 1050 pixels (display dot pitch 0.311 mm) at 120 Hz. The system latency was 24 msec, measured with a video-based method^40^, which is below the duration of a typical saccadic eye movement of the amplitude measured in the present study. The monitor was run from an NVidia Quadro FX 4600 graphics processing unit. Observers were seated 50 cm in front of the monitor with their heads stabilized in a chinrest and wore active stereoscopic shutter-glasses (NVIDIA 3DVision) during all experiments to control dichoptic stimulus presentation. The cross talk of the dichoptic system was 1% measured with a Spectrascan 6500 photometer.

### General Procedure

**Figure 1:**
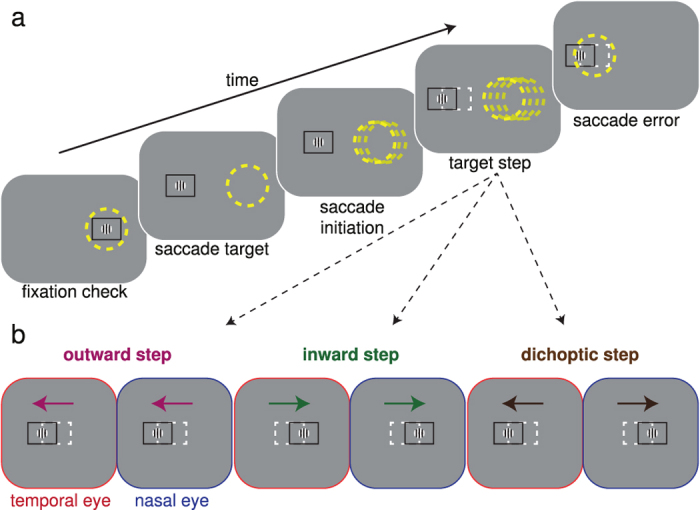
Saccade Adaptation Paradigm. (**a**) Schematic of a trial. The yellow dashed circle represents an observer’s hypothetical gaze location. The observer initially fixates a target Gabor, framed by a black nonius square to aid binocular fusion^41^. Once fixation is verified by the eye tracker, the target is shifted 8° leftward (or rightward across blocks), prompting the observer to make a leftward saccade. Saccade initiation is detected online (see Materials and Methods) and, during the saccade, the intra-saccadic target step displaces the target from its pre-saccadic position (dashed white box, not present in the stimulus). In this example, the saccade lands at the pre-saccadic target position, resulting in an oculomotor error signal. (**b**) Three experimental conditions. The intra-saccadic target step could be outward in both eyes (outward step), inward in both eyes (inward step), or outward in the temporally moving eye and inward in the nasally moving eye (dichoptic step).

**Figure 2:**
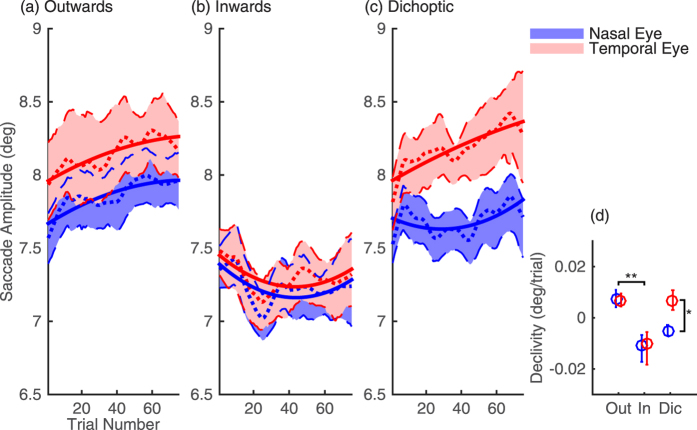
Saccade Adaptation. (**a**–**c**) Saccade amplitude for temporally (red) and nasally (blue) moving eye as a function of trial number for outwards (**a**), inwards (**b**) and dichoptic (**c**) conditions. Data are smoothed with a lowess regression with a span of 15 trials and averaged across sessions and observers (dotted lines). Shaded regions represent 68% bootstrapped confidence intervals of the mean. Solid lines are the average polynomial equations fitted to the data (see Text for details). (**d**) Declivity parameter of the polynomial fits estimated for each eye and each condition, averaged across sessions and observers. Error bars are 68% bootstrapped confidence intervals. *p < 0.05, **p < 0.001.

**Figure 3:**
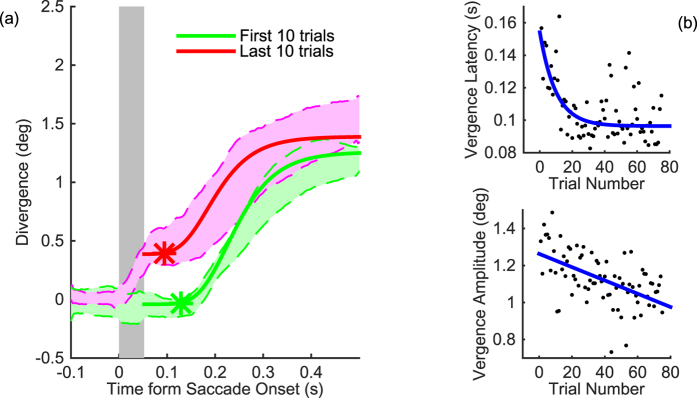
Independent Vergence and Saccade Adaptation: (**a**) Inter-ocular divergence around the time of a saccade following the dichoptic target step, averaged across observers and across the first 10 (green) and last 10 (red) trials. Grey shaded region represents the approximate period of the saccade. Red and green shaded regions are 68% bootstrapped confidence intervals of the median inter-ocular divergence as a function of time from saccade onset. Filled lines are the median logistic functions fitted to the data. Asterisks represent the point at which the vergence response is initiated. (**b**) Latency and (**c**) amplitude of the vergence response from saccade onset as a function of adaptation trial number. Data are the median for six observers. Solid blue lines are the best fitting exponential decay (latency) and linear (amplitude) functions passing through the data.

Schematics of our experimental design are shown in Fig. 1. An observer’s task was to make saccades directly to a Gabor target (σ = 0.5°, ω = 4 cycles/degree, 55% contrast) immediately after it stepped 8° to the left or to the right of initial central fixation. Each observer completed two sessions for each adaptation condition (outwards, inwards and dichoptic). In one session saccades were always to the left, in the other session saccades were always to the right. The order of conditions and sessions was counterbalanced across observers via a Latin square design. Within a session, an observer completed 175 trials: the first 25 trials were *Baseline Trials*, in which there was no intrasaccadic target displacement; trials 26 to 100 were *Adaptation Trials*, in which a different intrasaccadic shift was introduced depending on the condition; and trials 101 to 175 were *Recovery Trials*, in which the intrasaccadic target displacement was no longer presented.

At the start of each trial, subjects fixated the central Gabor target encompassed by a 4 × 3 (width × height) degree nonius bounding box. The nonius box served to aid vergence at the stimulus depth^41^. Subjects were required to make a correct Vernier judgment in order for the trial to commence (See Supplementary Experimental Procedures). This was performed to ensure that subjects were correctly fixating and attentive. Following the Vernier judgment, subjects were required to maintain fixation within the central 1.5° (with compliance monitored by the eye-tracker). After a random latency drawn from a uniformly distributed time interval (1000–1500 ms), the central Gabor was abruptly shifted 8° to either the left or right, depending on the session. Subjects were instructed to immediately saccade to the new eccentric target location and to maintain steady fixation on the eccentric target. After 1000 ms the target disappeared and the central red dot reappeared, indicating that a new trial could be commenced.

During the 25 *Baseline Trials and the 75 Recovery Trials*, the target shifted by 8°in both eyes with no further change in position. During the 75 *Adaptation Trials*, following the first 8° displacement, the target was displaced a second time by 0.8°. The direction of the secondary shift was outwards in both eyes (outwards condition), inwards in both eyes (inward condition), or outwards in the temporally moving eye and inwards in the nasally moving eye (dichoptic condition). This secondary target displacement took place as soon as the eye-tracker detected that the mean position of the two eyes had moved 2° from central fixation. The secondary displacement thus occurred during the saccade, ensuring that the secondary displacement was not detected^12^,^13^,^14^,^15^,^16^,^17^. After completing all experimental sessions (individual sessions were spread out over ~2 weeks of testing, each observer completed a total of 1050 trials), naïve observers were debriefed and in all cases were surprised to learn about the experimental manipulation: none had noticed the secondary displacement. During the dichoptic adaptation trials, all observers were aware that the end stimulus contained depth information, since the manipulation introduced a small amount of uncrossed disparity, even though they were unaware of the intrasaccadic displacement.

### Data Analysis

Saccades were detected via the Eyelink software in its default configuration, which classifies data samples as belonging to saccades when these exceed a velocity threshold of 30°/s, an acceleration threshold of 8000°/s^2^, and a motion threshold of 0.1°. Raw saccade amplitude data were smoothed with a lowess regression with a span of 15 trials for illustrative purposes. We fit the data with a second-degree polynomial (parabolic) equation (1):


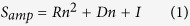


where *S*_*amp*_ is the saccade amplitude, *n* is the trial number, *R* is the rate of change of the parabola, and *D* is the declivity of the parabola at the y-axis intercept *I*. Saccade amplitude data and fitted parameter estimates were averaged across sessions and observers. Parameter estimates were analysed using a 2 (eye) × 3 (adaptation condition) within-subject ANOVA and, where appropriate, post-hoc analyses were performed using a Tukey–Kramer single-step, multiple comparison procedure. ANOVA normality assumptions were verified with Quantile-Quantile plots. A detailed breakdown of the results is shown in the Supplementary Data.

Vergence was computed with reference to the display screen as the difference in left and right horizontal eye coordinates (i.e., vergence was zero when both eyes were pointing at the same spot on the monitor surface). We removed from the vergence traces measured during the adaptation trials, the transient divergence typically observed during saccades^42^. This was accomplished by subtracting from the vergence traces measured during the adaptation trials the average vergence trajectory observed during the Baseline trials. Vergence data were then fitted with an asymmetric logistic function with equation (2):


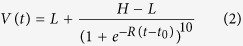


where *t* is time from saccade onset and *V*(*t*) is the vergence response. The amplitude of the vergence response was defined as *V*_*amp*_ = *H* − *L*. The time from saccade onset at which a vergence eye movement was commenced was estimated following equation (3) to be the time at which the vergence response had increased from baseline by 1/100^th^ of *V*_*amp*_:


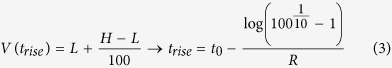


## Additional Information

**How to cite this article**: Maiello, G. *et al*. Monocular and Binocular Contributions to Oculomotor Plasticity. *Sci. Rep.* **6**, 31861; doi: 10.1038/srep31861 (2016).

## Supplementary Material

Supplementary Information
