# Supplementary material for: Monocular and Binocular Contributions to Oculomotor Plasticity
Source: Sci Rep. 2016 Aug 18;6:31861. doi: 10.1038/srep31861 (PMC4989160; doi:10.1038/srep31861)
Supplement: Supplementary Information [file srep31861-s1.pdf]

Supplementary Information to:

Monocular and Binocular Contributions to Oculomotor Plasticity

Guido Maiello[1,2,*], William J. Harrison[2,3,4], and Peter J. Bex[2]

[1] UCL Institute of Ophthalmology, University College London, 11-43 Bath Street, London EC1V 9EL, UK.

[2] Department of Psychology, Northeastern University, 360 Huntington Ave., Boston, Massachusetts 02115 USA.

[3] Department of Psychology, University of Cambridge, Free School Lane, Cambridge, CB2 3RQ, UK

[4] Queensland Brain Institute, The University of Queensland, St Lucia QLD 4072, Australia

[*] E-mail: guido_maiello@yahoo.it

Supplementary Data

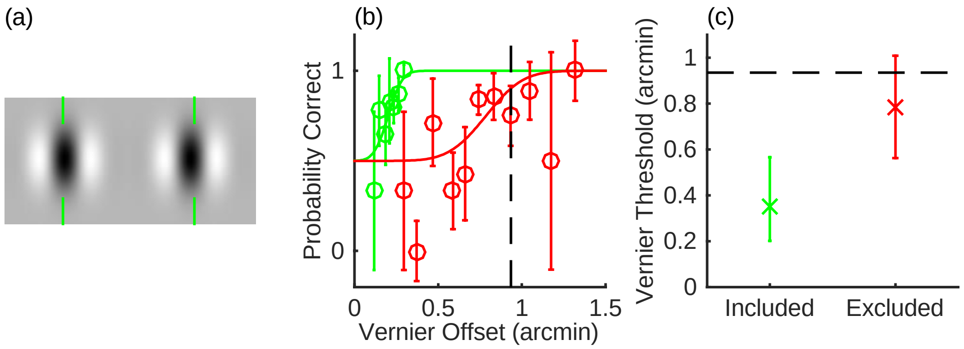

Figure S1: Stereoscopic Vernier Acuity. (a) Stimulus from a single trial (free-fuse the Gabor patches to view stereoscopically). Observers were required to fuse the stimuli, which were binocularly presented via shutter glasses, and report whether the vertical green lines were displaced to the left or to the right of the vertical dark Gabor bar. (b) Example data from one stereoscopically healthy naïve observer (green) and from the one observer who was excluded from this study for being stereo anomalous (red). The probability of a correct Vernier judgment is plotted as a function of line offset. Filled circles are the probability of a correct response at each offset level, error bars represent the binomial standard deviation. Sigmoid curves are best fitting cumulative normal functions. Vertical dashed line is the expected monocular performance and was therefore selected as the exclusion criterion for abnormal stereoscopic vision. (c) Vernier discrimination thresholds. Green cross is the mean threshold from the six observers included in the study. Error bars are bootstrapped 95% confidence intervals. Red cross is the estimated threshold for the excluded observer. Error bars are 95% confidence intervals of the fitted threshold. Horizontal dashed line is the threshold set to detect abnormal stereoscopic vision.

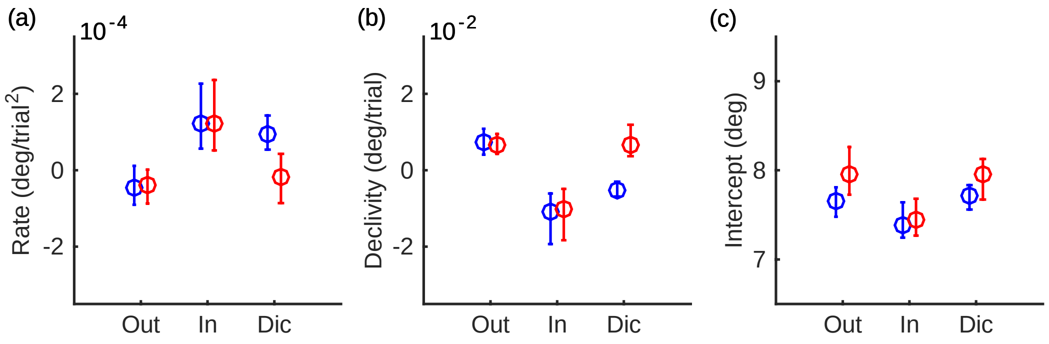

Figure S2: Fitted Oculomotor Adaptation Parameters. Rate (a), Declivity (b), and y-axis Intercept (c) parameters of the polynomial fits estimated for temporal (red) and nasal (blue) eye averaged across sessions and observers for each adaptation condition. Out and In refer to the conjugate adaptation conditions, Dic, refers to the dichoptic adaptation condition. Error bars are 68% bootstrapped confidence intervals.

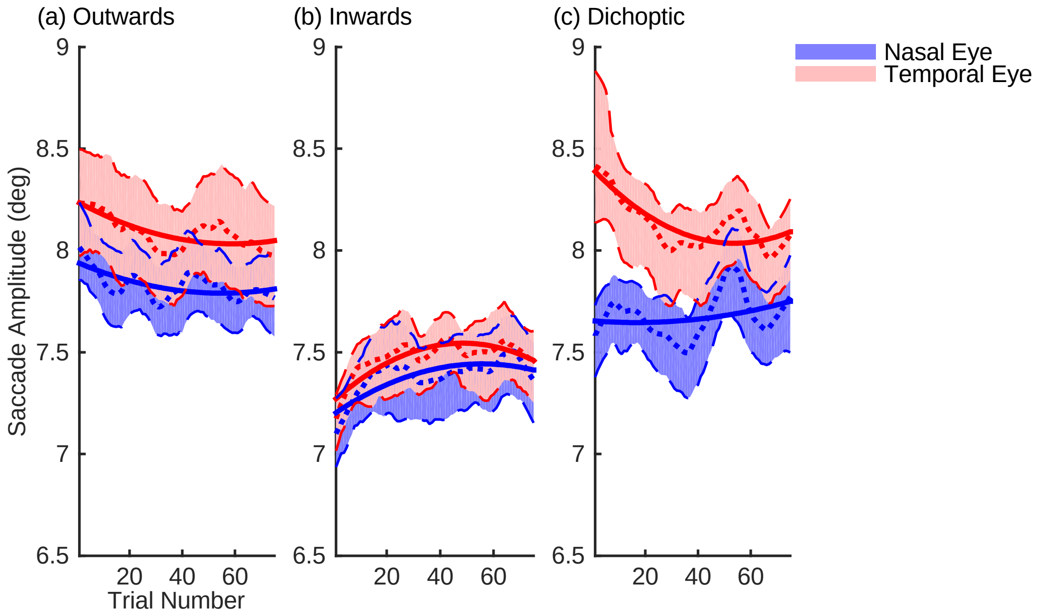

Figure S3: Recovery from Saccade Adaptation. (a-c) Saccade amplitude for temporally (red) and nasally (blue) moving eye as a function of trial number for outwards (a), inwards (b) and dichoptic (c) conditions. Data are smoothed with a lowess regression with a span of 15 trials and averaged across sessions and observers (dotted lines). Shaded regions represent 68% bootstrapped confidence intervals of the mean. Solid lines are the average polynomial equations fitted to the data.

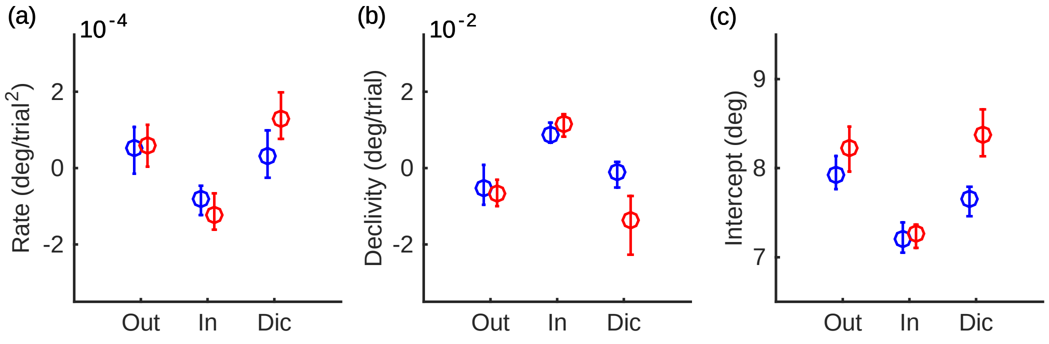

Figure S4: Fitted Recovery Parameters. As Figure S2, except for Recovery trials

Supplementary Experimental Procedures

Stereoscopic Vernier Acuity task.

Prior to the main experiment, observers were screened for normal stereo vision. This was accomplished by having observers perform a Vernier acuity task with stimuli displaced in depth (Figure S1a).

Observers were required to fixate a vertically oriented Gabor target (0.5° σ, 4 cycles/degree, 55% contrast) encompassed by a 4°x3° (WxH) nonius bounding box. The nonius box served to aid vergence at the stimulus depth [1]. Both fixation target and nonius box were rendered with 1.6° uncrossed disparity. If observers were binocularly fusing the stimulus, the Gabor target should have appeared ~14 cm behind the surface of the monitor. This was the same disparity observers would experience in the dichoptic adaptation experiment. Two vertical line segments, each 0.25° long, were presented directly above and below the Gabor target. On each trial, the segments were displaced to the left or right of the center of the Gabor target, at random across trials. The observers' task was to report via button press whether the line segments were to the left or to the right of the center of the Gabor target. The size of the displacement was under the control of two randomly interleaved 3 down 1 up staircases [2] that adjusted the Vernier offset to a level that produced 79% correct trials. The raw data from each staircase were combined and fitted with cumulative normal functions by least-squares regression, weighted by the binomial standard deviation. Vernier discrimination thresholds were estimated from the 75% correct point of the psychometric function. After verifying that the thresholds from the two separate fits agreed with each other, the raw data from the two staircases were pooled together to obtain a single threshold estimate. Example data and fitted functions from two observers are presented in Figure S1b.

Each trial, the line segments were randomly assigned 1.9 arcmin (4 pixels) of crossed or uncrossed disparity with respect to the disparity of the Gabor target. This meant that if the observers correctly perceived stereoscopic depth, the line segments would appear displaced in depth by 1 cm closer or farther to the observer with respect to the Gabor Target. If observers had abnormal stereovision and/or suppressed the visual input to one eye, the best performance achievable at the Vernier acuity task would be 0.9 arcmin (half the relative disparity between the Gabor target and the Vernier line segments). If observers could reliably perform better than 0.9 armin at the task, we took this as evidence that they were successfully combining information in the two eyes in order to make the Vernier judgment.

The mean Vernier threshold for all subjects included in the study is shown in Figure S1c. Six out of seven subjects exhibited Vernier thresholds that were indicative of good binocular summation. One subject performed the task at the level of monocular performance. When interrogated, the subject confirmed he had a two-line visual acuity difference between his left and right eye, consistent with mild amblyopia [3]. Thus, the subject was excluded from further testing.

Quantitative Investigation of Induced Saccade Adaptation

Saccade amplitude data from the adaptation procedure were fit to a second-degree polynomial (parabolic) equation:

$$S_{amp} = Rn^2 + Dn + I$$

where S_{amp} is the saccade amplitude, n is the trial number, R is the rate of change of the parabola, and D is the declivity of the parabola at the y-axis intercept I. For positive values of R the parabola opens upwards, whereas for negative values of R the parabola opens downwards. The declivity D is the slope of the parabolic curve at zero. For positive values of D the curve is increasing, for negative values the curve is decreasing.

The rate of adaptation R for temporally and nasally moving eye in each experimental condition, averaged across sessions and observers, is shown in Figure S2a. ANOVA results showed a significant main effect of adaptation condition ($F_{2,10}$=15.02, p= 0.00097). Specifically, the rate of adaptation was significantly greater in the inwards adaptation condition than both the outwards and dichoptic adaptation condition (p= 0.00071 and p=0.043 respectively). This is consistent with well-documented asymmetries between gain decreasing and gain increasing adaptation paradigms [4]. There was no main effect of adapted eye on the rate of adaptation (temporally moving eye vs nasally moving eye: $F_{1,10}$=2.22, p=0.17), and no significant interaction between eye and adaptation condition ($F_{2,10}$=2.53, p=0.13).

The declivity D (i.e. direction of adaptation) for temporally and nasally moving eye in each experimental condition, averaged across sessions and observers, is shown in Figure S2b. ANOVA results showed a significant main effect of adaptation condition ($F_{2,10}$=29.38, p= 0.000065), no main effect of adapted eye ($F_{1,10}$=4.51, p=0.057), and a significant interaction between eye and adaptation condition ($F_{2,10}$=4.62, p=0.038). The declivity was positive for the outward step condition and negative for the inward step condition (difference between outward and inward step conditions: p=0.000051). In both these conditions, there was no difference in the direction of adaptation between the temporally moving and nasally moving eyes (p=0.99). Conversely, in the dichoptic step condition, the direction of adaptation in the temporally moving eye was positive, whereas the direction of adaptation in the nasally moving eye was negative (difference between temporally and nasally moving eye: p= 0.036), confirming that the saccades in each eye were recalibrated according to the different error signal simulated in each eye.

The y-axis intercept I, i.e. the initial saccade amplitude, for temporally and nasally moving eye in each experimental condition, averaged across sessions and observers, is shown in Figure S2c. The initial saccade amplitude was larger for the nasally moving eye than the temporally moving eye for all conditions ($F_{1,10}$=7.75, p=0.019). We also observed a significant main effect of adaptation condition ($F_{2,10}$=13.5, p=0.0014), and no interaction between eye and adaptation condition

($F_{2,10}$=0.97, p=0.41). Note that these baseline differences in saccade amplitudes do not affect adaptation dynamics.

Quantitative Investigation of Recovery from Saccade Adaptation

Immediately after each run of adaptation trials, observers completed a recovery procedure in which they performed the same saccade task but without the intra-saccadic target step. Recovery patterns were the reverse of adaptation patterns for all three conditions (Figure S3), i.e. in both eyes adaptation gain decreased when recovering the outward step condition (Figure S3a), and decreased when recovering from the inward step condition (Figure S3b). When recovering from the dichoptic step condition (Figure S3c), saccade amplitudes decreased in the temporally moving eye and increased in the nasally moving eye.

Saccade amplitude data from the recovery procedure were fit to the same second-degree polynomial as per the adaptation trials.

The rate of recovery R for temporally and nasally moving eye in each experimental condition, averaged across sessions and observers, is shown in Figure S4a. ANOVA results showed a significant main effect of recovery condition ($F_{2,10}$=18.69, p=0.00042). The rate of recovery was significantly smaller in the inwards recovery condition than both the outwards and dichoptic recovery conditions (p=0.0017 and p=0.00058 respectively). There was no main effect of adapted eye on the rate of recovery ($F_{1,10}$=0.68, p=0.43), and no significant interaction between eye and recovery condition ($F_{2,10}$=2.33, p=0.15).

The declivity of the recovery curves for temporally and nasally moving eye in each experimental condition, averaged across sessions and observers, is shown in Figure S4b. ANOVA results showed a significant main effect of recovery condition ($F_{2,10}$=35.65, p=0.000028), no significant main effect of adapted eye ($F_{1,10}$=3.93, p=0.076), and a significant interaction between eye and recovery condition ($F_{2,10}$=5.7, p=0.022). The declivity was positive when the saccades were recovering from the inwards step condition and negative when the saccades were recovering from the outwards step condition (difference between outward and inward step conditions: p<0.001). In the dichoptic condition, the declivity of the temporally moving eye was clearly negative, while the declivity of the nasally moving eye was ~0 (difference between temporally and nasally moving eye: p=0.031).

The y-axis intercept of the recovery curves corresponds to the final saccade amplitude induced in each eye during the adaptation trials The y-axis intercept of the recovery curves for temporally and nasally moving eye in each experimental condition, averaged across sessions and observers, is shown in Figure S4c. ANOVA results showed a significant main effect of adapted eye ($F_{1,10}$=13.41, p=0.0044), with the temporally moving eye recovering on average from larger saccades. We also observed a significant main effect of recovery condition ($F_{2,10}$=29.66, p=0.000063) and no significant interaction between eye and recovery condition ($F_{2,10}$=3.8,

p=0.059). These data further confirm the success of the adaptation procedures in recalibrating saccade amplitudes.

Supplementary References

1 Shimono, K., Ono, H., Saida, S. & Mapp, A. P. Methodological caveats for monitoring binocular eye position with nonius stimuli. *Vision Research* **38**, 591-600 (1998).

2 Wetherill, G. & Levitt, H. Sequential estimation of points on a psychometric function. *British Journal of Mathematical and Statistical Psychology* **18**, 1-10 (1965).

3 Holmes, J. M. & Clarke, M. P. Amblyopia. *The Lancet* **367**, 1343-1351 (2006).

4 Pelisson, D., Alahyane, N., Panouilleres, M. & Tilikete, C. Sensorimotor adaptation of saccadic eye movements. *Neuroscience & Biobehavioral Reviews* **34**, 1103-1120 (2010).
